# Heart Failure: a Punch from the Gut

**DOI:** 10.1007/s11897-024-00648-y

**Published:** 2024-02-01

**Authors:** Ajay Mahenthiran, Jennifer Wilcox, W.H. Wilson Tang

**Affiliations:** 1 Case Western Reserve University, Cleveland, OH USA; 2 Department of Cardiovascular and Metabolic Sciences, Lerner Research Institute, Cleveland Clinic, Cleveland, OH USA; 3 Department of Cardiovascular Medicine, Heart Vascular and Thoracic Institute, Cleveland Clinic, 9500 Euclid Avenue, Desk J3-4, Cleveland, OH 44195 USA

**Keywords:** Heart failure, Gut microbiome, Microbial metabolites, Splanchnic vasculature

## Abstract

**Purpose of Review:**

This article seeks to elucidate the mechanisms underlying the bidirectional relationship between the gut and the heart, focusing on the pathophysiology of heart failure. We have previously demonstrated that Heart failure (HF) has significant effects on splanchnic vasculature and leads to key alterations in the gut microbiome, portending greater comorbidity with HF.

**Recent Findings:**

A growing field of research is focused on the effects of a “leaky gut” in the development of disease across organ systems. The leaky gut hypothesis centers on intestinal epithelial barrier dysfunction causing increased permeability of the gut and subsequent alterations to gut composition by endotoxins and microbial metabolites. Changes in the quantities of metabolites including short-chain fatty acids, trimethylamine *N*-oxide and other amino acid metabolites, and various bile acid species have been shown to result in gut dysbiosis and worsening HF.

**Summary:**

The gut plays a highly significant role in HF prognosis and requires greater attention for future therapeutic interventions. Treatments targeting gut composition could have very beneficial effects on HF prognosis.

## Introduction

Heart failure (HF) currently affects over 6.7 million adults in the USA alone, and the prevalence is expected to rise to 8.5 million by 2030 [1]. It is a complex clinical syndrome with signs and symptoms that result from a structural or functional impairment of ventricular filling or ejection [2]. Typical signs of HF include elevated jugular venous pressure, pulmonary crackles, and peripheral/abdominal edema, with typical symptoms including breathlessness and fatigue [2]. Additional non-cardiac complications of HF arise from the downstream effects on other systems and organs, particularly the gastrointestinal system [3]. Patients with HF often complain of abdominal symptoms, including early satiety, abdominal discomfort and swelling, and sometimes even abnormal bowel habits. However, asking about these symptoms has not been emphasized in the traditional bedside evaluation. This connection between the gut and HF has yet to be clearly defined. However, with the increasing prevalence of HF, there is a need to develop new comprehensive therapeutic approaches, and novel insights into the role of the gut in the pathogenesis and pathophysiology of HF may allow this connection to be exploited for more effective HF treatment [4]. As previously mentioned, the current understanding of the heart-gut axis is incomplete, and there remains a lack of understanding of how gut dysbiosis and other related metabolic disturbances in gut microbiota contribute to both HF disease progression and the promotion of risk factors such as atherosclerosis and hypertension [5]. Several gut microbial metabolic pathways, including the production of trimethylamine *N*-oxide (TMAO), phenylacetylglutamine (PAGln), and short-chain fatty acids (SCFAs), have been shown to play an important role in the progression of HF [4, 5]. In addition, the abdominal compartment (more specifically the splanchnic vasculature) may further contribute to hemodynamic derangements [6]. For example, increased ventricular filling pressures, a hallmark of HF, can co-occur with increased splanchnic congestion. Thus, there is important evidence that the abdominal region can serve an important role in the prognosis of HF. In this review, we aim to not only underline the physiologic consequences of HF on the gut but also the role the gut microbiome plays in HF pathophysiology.

## Physiologic Consequences of Impaired Hemodynamics on the Gut

Heart failure has been traditionally defined by impaired forward flow exemplified by a low cardiac output. From a hemodynamic standpoint, the problem has always been attributed to a forward failure caused by the heart’s inability to pump enough blood to the vital organs [7]. However, increasing evidence has shown a concurrent significant backward failure causing edema, leakiness, and engorgement of veins among other symptoms [6]. The inability of the ventricles to effectively pump out the blood entering them also increases ventricular filling pressures. This backward failure may produce a disproportionate arterial-venous blood distribution in the vasculature, leading to systemic venous congestion that impedes blood exiting the abdominal organs including the intestines, liver, and kidneys [8].

### Importance of Splanchnic Vasculature

Systemic congestion typically involves abdominal congestion, as the splanchnic vasculature holds up to 65% of the total blood volume in a typical circulatory system [9]. Three midline branches of the abdominal aorta supply blood to the abdominal organs: the celiac artery supplying blood to the foregut (stomach, spleen, and pancreas), the superior mesenteric artery to the midgut (pancreas, small intestines, colon), and the inferior mesenteric artery to the hindgut (colon). About 25% of cardiac output flows to the splanchnic system through these three main arteries. The hepatic portal vein also plays an important role in the splanchnic vasculature as 75% of splanchnic arterial flow reaches the liver only after moving through abdominal organs and entering the portal vein [10]. The remaining 25% of blood delivered to the liver gets there directly via the hepatic artery. Vascular channels (sinusoids) within the liver funnel both venous and arterial blood to the hepatic central veins, which then drain into the inferior vena cava and flow back to the heart. Volume recruitment from the splanchnic compartment to the systemic circulation is a key physiological response to stressors like physical activity and blood loss.

Within the abdominal vasculature, α_1_, α_2_, and β_2_ adrenergic receptors play an important role in congestive HF. When activated, α-adrenergic receptors cause vasoconstriction by constricting the hepatic arterial smooth muscle. β-adrenergic receptors cause vasodilation by decreasing vascular resistance and increasing blood flow through the hepatic artery. The α-adrenergic receptors are largely concentrated in the splanchnic arteries. The increased sympathetic stimulation results in catecholamine release causing vasoconstriction as the pressure in capacitance vessels rises [6]. With this progressive volume overload from splanchnic venous congestion, systemic congestion with increased intra-abdominal pressure (IAP) occurs [6]. In one study, 60% of patients with heart failure also had elevated IAP [11]. That study also importantly noted that persistently increased IAP despite diuresis has been associated with impaired renal function in congestive HF patients. Recent evidence suggests that modulating the greater splanchnic nerve may impact excess fluid redistribution, thereby potentially improving cardiac filling pressures and exercise capacity [12•].

The spleen is another organ that acts as an important part of the splanchnic vasculature, receiving about 5% of cardiac output [13]. Blood flow through the spleen modulates splanchnic congestion as the splenic vein joins with the superior mesenteric vein to form the portal vein [13]. Splenic contraction may provide an incremental increase in systemic blood volume during exertion especially in the setting of severe cardiac impairment. With increasing splanchnic congestion in HF, the microvascular pressure within the spleen tends to increase, and fluid flows out of the spleen, thereby overloading the lymphatic system and leading to interstitial edema [6, 13].

### Inflammatory Hypothesis of Heart Failure

**Fig. 1:**
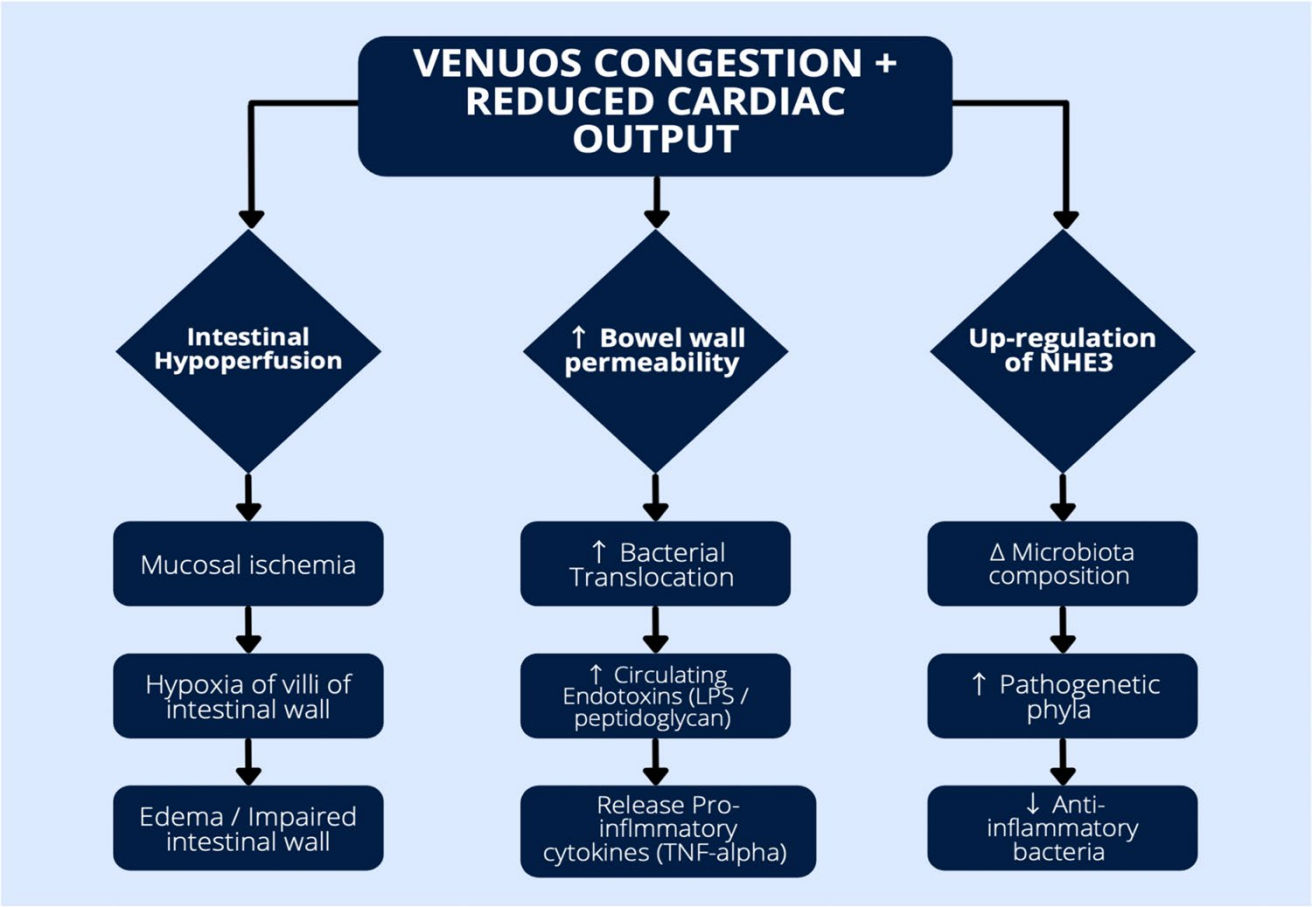
Pathophysiological mechanisms behind the “gut hypothesis” of HF, particularly the association of splanchnic hemodynamics with disruption of the intestinal wall and gut dysbiosis

Hemodynamic derangements in HF can directly disrupt intestinal mucosa structure, with impaired cardiac output associated with intestinal edema, ischemia, and inflammation (Fig. 1) [4]. Specifically, venous congestion and splanchnic ischemia in HF may induce bowel hypoperfusion, leading to hypoxia of the villa of the bowel wall [14]. Disruption of this intestinal mucosal barrier leads to intestinal permeability and resultant translocation of gut bacteria and microbial products into the circulatory system (so called “leaky” gut) [14]. The diffusion of bacterial endotoxins and inflammatory cytokines into systemic circulation can activate the inflammatory response that is characteristic of HF [14]. Besides inducing systemic proinflammatory cytokines, endotoxins can also lead to an intracardiac inflammatory response that directly damages cardiomyocytes. For example, lipopolysaccharide (LPS) is a well-known endotoxin that diminishes ventricular contractility by binding to toll-like receptor 4 (TLR4) on cardiomyocytes.

Abnormal sodium and fluid handling in the gut can co-occur with gut dysfunction caused by hypoperfusion. Sodium-hydrogen exchanger 3 (NHE3) is a heavily regulated electrolyte channel within the gut involved in regulating the exchange of sodium and protons. NHE3 is crucial for maintaining the balance between sodium secretion into, and absorption from, the gut. Proper NHE3 functioning is essential to maintaining salt, volume, and acid-base homeostasis. In congestive HF, the function of the NHE3 channel is upregulated because of hypoxia from venous congestion [15]. As a result, reabsorption of Na^+^ increases, and the subsequent increase in fluid absorption generates an unusual salt and water load on the cardiovascular system, worsening heart dysfunction. Therefore, NHE3 inhibition in the intestine can result in luminal sodium and water retention, leading to reduction in intestinal sodium and phosphate absorption and increased stool excretion, and even lower blood pressure [16]. Indeed, novel NHE3 inhibitors such as tenapanor have been used to treat hyperphosphatasemia in chronic kidney disease, while inhibitory properties of sodium glucose cotransporter-2 (SGLT2) inhibitors on NHE3 may explain some of their well-established benefits in the heart failure population [17].

## Gut Microbial Contributions to Metabolic Aspects of Heart Failure

The gut luminal hypoxia that can occur in HF patients can significantly affect the composition of the commensal gut microbiota. A common theme in the gut microbiome of patients with HF is a shift to pathogenetic phyla and a diminished number of bacteria with anti-inflammatory properties. A study by Pasini et al. indicates that, in comparison to healthy controls, HF patients had significantly increased quantities of pathogenic bacteria such as *Candida* [18]. Additionally, it was found that the extent of colonization by pathogenic bacteria like *Clostridium difficile* correlates with HF severity. These observed patterns of gut microbial dysbiosis in HF have also been noted in other cardiovascular diseases like coronary artery disease and in chronic systemic diseases like type 2 diabetes. Importantly, a common symptom of these conditions is a state of inflammation, which is often associated with a disrupted gut microbiome.

**Fig. 2:**
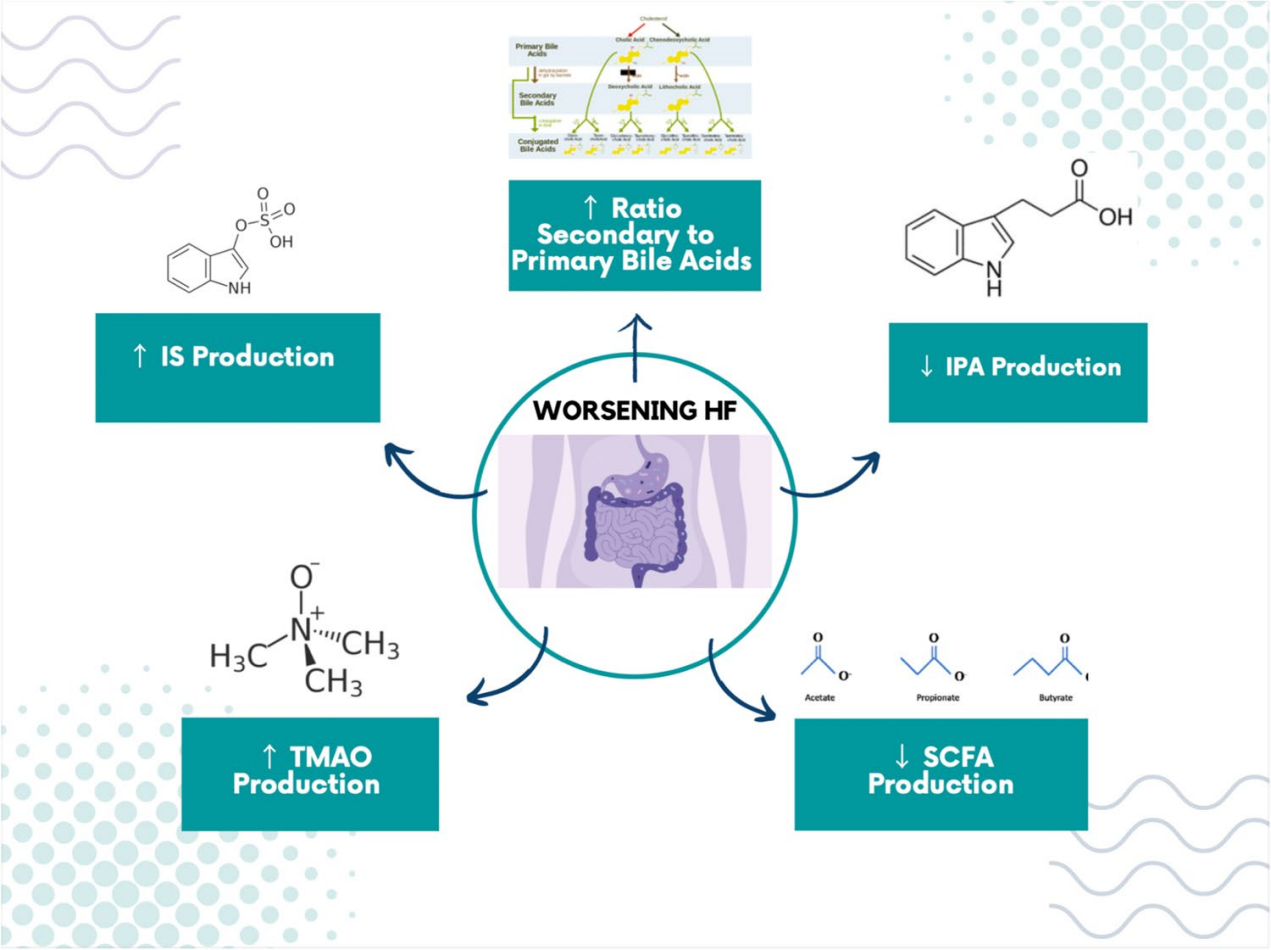
Gut microbial metabolites with active roles in HF pathophysiology. The metabolites fill important roles in susceptibility to HF and have been linked to adverse outcomes over time. The metabolites represent biomarkers and potential avenues for future therapeutics in treating HF

The gut microbiome’s role as a contributor to HF pathogenesis and adverse outcomes has increasingly been suspected, but delineation of precise pathways and participants involved is unclear. The gut microbiome produces metabolites that can be readily absorbed into the host bloodstream, which likely play a key role in mediating the inflammatory state characteristic of HF (Fig. 2). Approximately 10% of circulating small metabolites in mammalian blood is gut microbiome-related, implicating the gut as a significant environmental factor in human diseases [19]. Using metabolic approaches to better characterize the nature of these gut metabolites during HF can provide significant findings to better guide future diagnosis and therapy.

### Short-Chain Fatty Acids

Short-chain fatty acids (SCFAs) are the result of fermentation of indigestible nutrients like fiber and complex carbohydrates by the gut microbes. They act on several different G-protein coupled receptors, allowing them to play an essential role in regulating fluid and electrolyte homeostasis along with maintenance of the epithelial barrier. Common SCFAs like acetate, butyrate, and propionate act as protectors of intestinal mucosa [20, 21]. Butyrate and propionate specifically aid in regulating blood pressure and exhibit anti-inflammatory effects through T-cell activation. Butyrate also plays an essential role in regulating the integrity of the intestinal barrier and suppressing intestinal and extra-intestinal inflammation. Not surprisingly, it has been demonstrated that depletion of these SCFAs, and known SCFA producers, is associated with disruption of the intestinal barrier and dysbiosis of gut microbiota in HF patients. Using 16S ribosomal RNA sequencing in fecal samples, Kamo et al. demonstrated that patients with HF have an increasingly depleted number of SCFA-producing gut microbes [22].

Not only are SCFAs needed for intestinal barrier function, but they also have a cardioprotective role. Kaye et al. demonstrated that mice fed a diet lacking in prebiotic fiber were prone to hypertension and cardiac hypertrophy, but when SCFAs were introduced to these mice, with no other alterations to their diet, these phenotypes were no longer displayed [23]. However, despite the demonstrated beneficial role of SCFAs, there are still debates regarding the therapeutic use of SCFA supplementation. Some studies have shown that neither acetate nor fiber could change cardiac remodeling and override the development of HF [24, 25•].

### Trimethylamine N-oxide

Trimethylamine *N*-oxide (TMAO) is a gut microbiome-mediated metabolite that has been identified as a biomarker for cardiovascular disease and provides a well-characterized example of gut-host interactions with downstream effects in HF [26]. Dietary nutrients containing a trimethylamine (TMA) moiety, including choline, phosphatidylcholine, and carnitine, can be metabolized by the gut microbial enzyme TMA lyase, which cleaves the TMA from the parent nutrient. Once cleaved, the free TMA in the intestinal lumen can be absorbed into hepatic circulation where host enzymes, particularly hepatic flavin monooxygenase 3 (FMO3), convert TMA into TMAO [26].

TMAO has a multitude of demonstrated negative effects on the cardiovascular system. For example, high TMAO levels have been linked to atherosclerotic heart disease and overall major adverse cardiovascular events (MACE) [27, 28]. Murine studies have demonstrated that mice treated with supplemental TMAO or choline before HF development saw worsening pulmonary edema and increased systolic dysfunction. However, these effects were reversed with dietary changes or inhibition of microbial enzymes generating TMA/TMAO [29, 30]. However, the exact TMAO host receptor through which TMAO promotes HF is still unknown. Elevated TMAO levels are common in HF patients and correlate with diastolic dysfunction, which may mean that venous congestion plays an important role in altering the gut microbiome to increase TMAO production. Such a finding would be of immense significance considering that elevated baseline TMAO levels have been implicated as a predictor of mortality in patients with HF [31].

### Amino Acid Metabolites

Gut bacteria play an essential role in fermenting and metabolizing specific dietary amino acids (AA) such as phenylalanine, tryptophan, and tyrosine. Tryptophan is metabolized into indole which can then be converted into either indole-3-propionate (IPA) or indoxyl sulfate (IS). Indoles like IPA play an important role in maintaining the integrity of the intestinal wall, reducing both permeability and the entering of circulating inflammatory cytokines [32]. It has been postulated that IPA acts as a mitochondrial modulator in cardiomyocytes and has been shown to alter cardiac function in an *ex vivo* mouse model [33]. As is true for most microbial metabolites, only certain bacteria in the gut microbiome can metabolize amino acids to make IPA. Hence, changes in the composition of the gut microbiota could easily result in decreased IPA levels. A result of this shift in composition is increased permeability of the intestinal wall and the mechanisms that describe the “gut hypothesis” of HF.

Although microbial metabolism of dietary AAs can produce beneficial downstream effects, other effects can be harmful. As previously mentioned, tryptophan can be converted into IS instead of IPA, and tyrosine and phenylalanine can be metabolized into p-cresyl sulfate (pCS). Both IS and pCS are uremic toxins that exhibit biological toxicity on the kidney and cardiovascular system [34]. Furthermore, IS specifically has demonstrated adverse cardiac effects in cells. In a human monocytic cell line, treatment with IS increased expression of TNF-ɑ and IL-1β, two pro-inflammatory cytokines that stimulate adverse cardiac remodeling including hypertrophy and collagen fibrosis [35]. Another study demonstrated that high IS levels were associated with cardiac dysfunction, particularly diastolic dysfunction, and a heightened risk of cardiac events in patients with dilated cardiomyopathy [36]. Therefore, a therapeutic benefit may be obtained by targeting the gut microbial enzymes that generate pCS and IS to reduce or inhibit the pCS/IS-mediated pro-thrombotic phenotype [37•]. Removal of uremic toxins from the GI tract, before they are systemically absorbed, also has the potential to be an effective treatment. Previous studies have demonstrated that oral uremic toxin adsorbents can reverse HF by stopping myocardial apoptosis [38].

Phenylacetylglutamine (PAGln), a meta-organismal metabolite derived from microbial fermentation of phenylalanine to phenylacetic acid followed by glutamine conjugation by host liver enzymes, has also been linked to adverse cardiac events in patients with chronic kidney disease [39]. PAGln fosters heightened thrombotic risks in animal model studies and in human platelet studies, which likely accounts for the observed association between elevated PAGln levels and clinical thrombotic event risks [40]. Moreover, the effects of PAGln, which possesses structural similarity to catecholamines, were shown to be mediated via adrenergic receptors [40]. In addition, our group recently reported a striking association between PAGln levels and prevalent HF, as well as the ability of PAGln to foster a negative inotropic effect on both epinephrine-stimulated isolated cardiomyocytes and stimulation of the gene responsible for cardiomyocyte natriuretic peptide expression *in vitro* and *in vivo* [41•]. In patients with HF, plasma PAGln levels served as an independent predictor of an increased risk of adverse cardiovascular events in HF that was complementary to NT-proBNP levels [42•, 43].

### Bile Acids

Primary bile acids (BA) are synthesized in the liver through the oxidation of cholesterol. BAs are actively secreted into the bile along with cholesterol and phospholipids and play an essential role as emulsifiers through the absorption of lipids and fat-soluble vitamins. Primary BAs are predominantly recycled and reabsorbed in the ileum via the hepatic portal vein in the enterohepatic cycle. Those that are not recirculated to the liver end up in the colon where the gut microbiota use bile salt hydrolases to convert them into secondary BAs [44].

BA studies have shown contradicting effects on cardiac structure and function. In the past, it was thought that primary BAs caused only negative inotropic and chronotropic effects [45, 46]. Recent studies have shown that bile acids can have varying effects depending on which BA receptor is expressed on the cardiomyocyte. For example, one study demonstrated that BAs binding to Takeda G protein-coupled receptor 5 (TGR5) improved myocardial survival following physiologic and hemodynamic stress in mice [47]. However, another investigation demonstrated that BAs binding to a different receptor, the farnesoid X receptor, resulted in cardiac apoptosis and contributed to myocardial ischemic injury [48].

The types of BAs present in one’s body differ between healthy individuals and individuals with chronic HF, in part because of gut dysbiosis. Most notably, Mayerhofer et al. found a higher ratio of secondary BAs to primary BAs in patients with chronic HF [49]. Although this may lead to an assumption that primary BAs are more beneficial for individuals, a separate study indicated that secondary BAs can also have positive effects in reversing HF [50]. This study, where patients with HF were treated with the secondary BA ursodeoxycholic acid, found that the BA exerted anti-inflammatory properties and improved peripheral blood flow by trapping LPS, an inflammatory cytokine, in micelles. These contrasting findings suggest that since individual BAs have varying effects on HF patients, the development of HF may be associated more with an imbalance of bile acids rather than a specific, or group of specific, BAs.

### Conclusions and Future Direction

Heart failure remains an immense health burden in the USA despite the plethora of treatment regimens now available as many of those regimens have proven ineffective. Recent studies have demonstrated the impact of hemodynamic derangements on gut physiology and the important subsequent contribution to the development and prognosis of HF. Future studies analyzing host cardio-abdominal-renal interactions are warranted, especially in the context of continuous congestion. Understanding more about splanchnic blood vessels and microcirculation may also offer alternative diagnostic routes for HF, and current clinical trials are investigating promising new drug (e.g., NHE3 inhibitors) or device (e.g., splanchnic nerve ablation or renal denervation) therapies for relieving splanchnic congestion [51].

Importantly, a growing body of evidence demonstrates that alterations in the gut microbiome may serve as a mediator in furthering the pathogenesis of many of the complications that arise from HF. Specifically, intestinal barrier breakdown, microbial translocation, and an altered composition of the gut microbiome have all been shown to have an immense impact on HF pathogenesis. Furthermore, with increased investigation of microbial metabolites like SCFAs, TMAO, BAs, and amino acid metabolites, we have been able to increase our understanding of the intricate host-microbe interactions in HF. These metabolites also serve as promising therapeutic targets and, as we move into an era of personalized medicine, measuring an individual’s specific metabolite levels could help direct personalized pharmacologic or dietary interventions.
